# CHLA 2026 CONFERENCE POSTERS / ABSC CONGRÈS 2026 AFFICHES

**DOI:** 10.29173/jchla29969

**Published:** 2026-08-03

Caitlyn Ford, Robyn Butcher, Amanda Hodgson, Emina Jasarevic, Madeline Hunter, Flynn Vipond Canada's Drug Agency

**Introduction:** The Research Information Services (RIS) team at Canada’s Drug Agency (CDA-AMC) has created an internal Archive of reports authored or published by the organization. The goal of this Archive is to make reports discoverable and available to staff in one central repository, providing access to full text reports, regardless of date published, or where the files have been stored. **Description:** A team of 3 librarians, 1 library technician, and 1 library technician intern (student) collaborated with our web team and project management office to develop a list of all projects produced by CDA-AMC. The Archive was built within the existing library catalogue. A customized Metadata Standard was created which focused on linked, keyword-based description, standardized material types, and includes special fields that focus on internal identifiers, such as project codes. To ensure the Archive meets the needs of staff, a test and learn approach was undertaken with volunteers tasked to find a sample of reports and asked how they achieved their results. These learnings were then incorporated into the living Metadata Standards document. **Outcomes:** The Archive already includes over 2000 project records with continual additions providing historical, full text access to CDA-AMC work. **Discussion:** This project highlights the success of a team of librarians and library technicians working together, bringing archives into a special library with a records management lens. Establishing a consolidated archive enables all staff to search, browse, and retrieve our reports, optimally leveraging previous work and enhancing our workflows and information systems.

