# Supplementary material for: CHLA 2026 CONFERENCE POSTERS / ABSC CONGRÈS 2026 AFFICHES
Source: J Can Health Libr Assoc. 2026 Aug 3;47(2):90–5. doi: 10.29173/jchla29969 (PMC13590139; doi:10.29173/jchla29969)
Supplement: Related texts

# Abstract: PP2. Developing a validated search filter to locate studies on sexually transmitted and blood-borne infections

Karly Gunson^1^, Erin Langman^1^, Kristy Hancock^2^, Heather Tornblom^1^

^1^University of Saskatchewan, ^2^University of Northern British Columbia

**Introduction:** Sexually transmitted and blood-borne infections (STBBIs) are a class of communicable illnesses caused by bacterial, viral, and other parasitic organisms. The ability to effectively search for literature is critical for evidence synthesis and evidence-based practice, but lack of a standardized definition and variability in terminology contribute to the “search-resistant” nature of STBBIs as a topic. We will address this challenge by creating and validating a search strategy for STBBIs. **Methods:** To limit scope, a list of six sexually transmitted infections targeted by the Public Health Agency of Canada was used to operationalize the definition of STBBIs. Based on this definition, a gold standard set of references was developed from studies on STBBIs retrieved from Ovid Medline. Text mining and term analysis were used to assess subject coverage and ensure balanced representation of each infection. Gold standard references were divided into a development set to inform search filter construction, and a validation set for testing search filter effectiveness. **Results:** This poster presents preliminary results from the development of the STBBI search filter, focusing on key decisions, challenges encountered, and approaches used to address them. It also outlines planned next steps for further development and validation. **Conclusion:** This validated search strategy will help address the search-resistant nature of STBBIs and support researchers and clinicians with identifying relevant literature for evidence synthesis and evidence-based practice. Thoughtful planning, clear definition of scope, careful selection of terminology, and iterative refinement are essential for overcoming the challenges of creating validated strategies for STBBIs and other search-resistant topics.

# Abstract: PP3. Guide Michelin de l’analyse de citations : outils gratuits assistés par IA vs. Web of Science (WoS)

Thiên Sa Hoàng^1^, HongYue Pan^2^

^1^Université de Montréal, ^2^Institut national de psychiatrie légale Philippe-Pinel

De plus en plus de chercheur(e)s rapportent utiliser un outil d’intelligence artificielle (IA) pour découvrir la littérature ou pour leur synthèse des connaissances (SC) (Langue et al., 2024; Oxford University Press, 2024; Segooa et al., 2025; van Genk et al., 2023). Research Rabbit (RR), un outil basé sur l’IA, propose de s’inscrire dans le flux de travail des chercheurs en facilitant l’accès aux références, citations et travaux similaires. The Lens, quant à lui, utilise également l’apprentissage automatique pour rendre accessibles, entre autres, les données de citation. Jusqu’à présent, l’outil de référence pour des analyses de citations était Web of Science (WoS), une propriété de Clarivate. Notre projet consiste à comparer la performance de cet outil traditionnel et établi (WOS) avec deux outils IA disponibles en accès libre, soit RR et The Lens. Nous réaliserons une analyse des références d’un échantillon d’articles aléatoires afin d’évaluer la fiabilité des outils IA et d’identifier d’éventuelles sources d’erreur. Pour les trois outils, nous calculerons le pourcentage de « véritables » références et de références manquantes par article. Enfin, nous évaluerons la valeur ajoutée de chacun des outils en termes de qualité des métadonnées et d’option de téléchargement des références. Nous espérons que notre collecte et analyse de données pourront proposer des points d’amélioration à ces outils gratuits, et ainsi augmenter leur potentiel pour des synthèses des connaissances.

# Abstract: PP4. Vendor trading cards: a whimsical guide to health sciences resources

Amanda Yanke

Health Knowledge Network

**Background:** Health sciences librarians navigate a complex vendor landscape that includes databases, journals, and point-of-care tools. New librarians or those unfamiliar with health sciences resources may find it challenging to quickly assess key vendors and their offerings. Orientation tools that simplify this process can enhance confidence and efficiency. Visualizations are already used in the healthcare sphere to empower patients to be more involved in their own care (Sakarang, Asli, & Hamzah, 2025). **Objective:** This poster introduces a visually engaging resource that introduces major academic vendors and their products through a series of infographic “trading cards,” inspired by the Publication Facts Label concept from the 5-Point Journal Integrity Initiative (Willinsky and Pimentel, 2024). **Methods:** Vendor profiles are compiled using publicly available information from company websites, product documentation, and accessibility statements. Each infographic includes standardized “fast facts,” such as founding year, organizational type (public, private, non-profit), flagship products, resource categories (databases, journals, point-of-care tools), and applicable health disciplines. Additional data points include COUNTER compliance, accessibility commitments (e.g., VPATs), and mission statements. Design emphasizes clarity and visual appeal, drawing on the aesthetics of sports trading cards. **Results:** The poster will describe the background and methods, and showcase examples of infographics suitable for use as an orientation tool for librarians new to health sciences resources. These will provide quick, comparable insights into vendor characteristics and offerings. **Conclusion:** This project combines a whimsical design approach with practical utility, supporting professional development and resource literacy among health sciences librarians. Future directions include expanding the set of vendors and exploring interactive digital formats.

**References:**

1. Sakarang, S. F., Asli, M. F., & Hamzah, M. (2025). Narrative visualization: Taxonomy, methodologies, challenges, and opportunities. Information Visualization, 24(2), 199-223. https://doi.org/10.1177/14738716251315916 (Original work published 2025).
2. Willinsky, J. and Pimentel, D. (2024), The publication facts label: A public and professional guide for research articles. Learned Publishing, 37: 139-146. https://doi.org/10.1002/leap.1599.

# Abstract: PP5. API-driven workflows for evaluating Open Access and APC savings in health libraries

Chloe Thierstein, Oriane Edwards

University of Toronto Libraries

Gathering, processing, and analyzing bibliometric data is often time-consuming and resource intensive, yet the growing availability of Application Program Interfaces (API) offers a promising solution. An area of importance to health libraries is Open Access (OA) publishing, especially as the Canadian Tri-Agencies pivot towards implementing open access publishing requirements within their funding packages. In this evolving publishing landscape, health librarians must ensure that decisions around OA publishing agreements, Article Processing Charge (APC) discounts, and collections budgets align with institutional and funder priorities. This poster explores how health sciences libraries can leverage automated, API-driven data collection workflows to analyze institutional publishing patterns such as OA publishing, authorship trends, journal output, and APC savings. Utilizing data directly from the Scopus API and the OpenAlex API, libraries can more effectively evaluate the performance and value of read-and-publish agreements or OA discount programs. We leveraged these APIs to gather and analyze data for the University of Toronto’s Faculty of Dentistry’s 2024 publications. Through our case study, we investigated the application of these tools for analyzing the Faculty’s OA publishing trends, comparing the capability of these tools for collecting key bibliometric indicators, such as research output volume, OA status, journal metrics, and savings from OA agreements. We highlight strengths and weaknesses, emphasizing data coverage, metadata quality, and integration ease. We assess how these automated workflows reduce manual labour, minimize errors, and improve reproducibility. Finally, this poster highlights the potential of incorporating these emerging tools to effectively respond to bibliometric challenges in contemporary health librarianship.

# Abstract: PP6. Developing a systematic review calculator, and the secondary studies that resulted from analyzing over 7,000 systematic reviews

Nicole Askin^1^, Tyler Ostapyk^1^, Orvie Dingwall^2^, Carla Epp^1^, Gail Matheson^2^

^1^WRHA Virtual Library, ^2^University of Manitoba

**Introduction:** Systematic reviews are recognized as the gold standard of scientific evidence and require authors to follow comprehensive and transparent methodologies. The ongoing primary study aims to develop a calculator to estimate time/resource investment based on preliminary PubMed/Medline results, while several secondary studies will evaluate how well published reviews adhere to established methodological standards and provide guidance for practitioners and journal editors. **Methods:** A corpus was collected by searching Scopus for articles published in 2023 that cited PRISMA 2020. Nearly 19,000 unique articles were imported into Covidence, of which 14,779 self-identified as a systematic review and/or meta-analysis. Two subsets were derived from this dataset for further analysis: 485 articles which included PubMed/Medline but no other databases, and 7,352 articles which included PubMed/Medline, at least one other database, and a numerical breakdown of results by specific database. **Results:** The analysis of these two datasets will be used for several purposes: Develop a calculator that practitioners can use to estimate total capture, screening time, and other metrics based on their preliminary PubMed or Medline search; Provide a snapshot of which databases and data sources are being searched in published systematic reviews, to provide guidance for searching and collection development; Describe features of reviews which claim to be systematic yet search no databases beyond PubMed/Medline; Lament the extraordinary variety of errors and methodological flaws reflected in published reviews, as a cautionary tale for journal publishers and reviewers.

# Abstract: PP7. Modernizing EndNote support services at Canada's Drug Agency

Danielle MacDougall, Hannah Loshak, Caitlyn Ford, Robin Featherstone

Canada's Drug Agency

**Introduction:** The EndNote Support Team with Research Information Services at Canada’s Drug Agency has customized a suite of online training materials for using EndNote at the organization. Our goals were to modernize EndNote support services for asynchronous learning, to empower employees transitioning to AMA 11 reference style, and to ensure high-quality referencing in our reports. **Description:** In response to a 2022 organizational survey indicating a preference for asynchronous learning tools, a team of 3 librarians and 1 library manager collaborated to develop a suite of multimedia “Endnote 21 Resources” to appear in the “Employee Essentials” section of our organizational SharePoint site. Team members used institutional software (e.g., Clipchamp) to create training videos and help sheets for all employees. Materials covered topics such as how to set up EndNote for use at Canada's Drug Agency, how to add references to manuscripts using a custom AMA 11 style, and how to use EndNote while multiple authors are working on the same reports. **Outcomes:** An initial suite of EndNote 21 Resources included 3 training videos, 3 guides, and 8 help sheets. They are used to train new staff, support complicated EndNote tasks (e.g. travelling libraries), and to provide troubleshooting support for the most common issues (e.g. disabled Endnote Toolbars). New materials are added when a need is identified. An evaluation survey will be conducted a year from the launch of the resources. **Discussion:** Our proactive approach to leverage Microsoft 365 utilities empowered employee competencies with Endnote through on-demand learning tools.

# Abstract: PP8. Building awareness of paper mills: an open educational approach

Kaitlin Fuller^1^, Erica Nekolaichuk^2^, Alison Manley^3^, Catherine Gracey^4^, Patricia Ayala^2^

^1^St. Francis Xavier University, ^2^University of Toronto, ^3^Saint Mary’s University, ^4^University of New Brunswick

**Introduction:** Paper mills are a growing problem in the world of scholarly publishing. A multi-institutional team of librarians got together to locate resources to share with our learnings to increase awareness of this issue. Though we found that awareness of these organizations is growing amongst researchers, we were unable to locate sufficient education resources designed for graduate students and early career researchers on paper mills. The team decided to build an open educational resource that would be specifically for early career researchers and could also be used by faculty and librarians. **Description:** We built an interactive module using the Articulate Rise platform and designed it to be freely available online. The module will consist of four sections introducing learners to paper mills: 1) Paper mills and how they work 2) How did we get here? 3) Community level responses 4) Tips for researchers 5) Tips for readers. Feedback from previous discussions with graduate students about paper mills indicated students were feeling overwhelmed and helpless; therefore, we prioritized keeping the tone conversational and focused the content on actionable strategies. **Outcomes:** Community feedback from upper-level undergraduate students, graduate students, librarians, and faculty shaped substantial revisions to the module. Based on this feedback, the following changes were made: researcher and reader tips were separated into distinct sections; interactive content was expanded throughout; horizontal sliders were removed; and overall writing clarity and content were improved. **Discussion:** Paper mills are a significant problem for researchers, both as producers of knowledge who may be unwittingly involved in paper milling operations, and as consumers of knowledge using an evidence base that is under threat. Librarians are well-positioned to take up the role of raising awareness of paper mills.

# Abstract: PP9. AI-deas for responsible use of AI in clinical research, scholarly works, teaching and learning

Stacy Posillico, Kristin Chapman, Janice Lester, Jaclyn Morales, Brendan Plann-Curley, Mary Shah, Regina Vitiello

Northwell Health

**Introduction:** As students, trainees, and early-career clinicians rapidly adopt generative artificial intelligence (AI) tools in their learning and research workflows, faculty and program leaders seek guidance on modeling and teaching ethical, effective generative AI use. Our Library team created educational sessions to meet this need and equip educators to identify, apply, and guide responsible use of AI-based tools in scholarly and research activities. **Description:** The Library team offers comprehensive educational programming online in synchronous sessions throughout our healthcare system. At hospitals, we provide one-hour interactive sessions on generative AI fundamentals, associated risks of use, proper prompting techniques, and useful AI platforms that we use in our own work. At a system-wide faculty development event, we presented a gamified, case-based exercise to identify teachable moments and develop mentoring approaches for trainees using AI in scholarly activities. Our research institutes hosted our first workshop on responsible generative AI use for literature searching, manuscript improvement, and evolving ethical best practices in scientific publishing. All sessions emphasize data privacy and protection, critical analysis of AI output, bias mitigation, environmental sustainability, and opportunities for librarian partnership in navigating this emerging landscape. **Outcomes:** Evaluations demonstrate increased competencies among educators in using AI responsibly for teaching, learning, and mentoring activities. Faculty report enhanced confidence in integrating AI tools while addressing ethical considerations. **Discussion:** Health sciences librarians can effectively empower faculty and leadership to maximize generative AI tool benefits, mitigate application risks, and cultivate AI competencies for themselves and their learners throughout their research and scholarly activities.

# Abstract: PP10. Organized, united, successful: student-led change in access to medical education support resources

Kelly Thormodson, Jeff Wagner

Penn State University

**Introduction:** Libraries struggle to communicate with key user groups such as students regarding resources needed to prepare for medical school tests and board exams. After needing to cancel a well-liked educational resource because of cost, students were disappointed. **Description:** Libraries often face challenges in effectively communicating with key user groups, particularly students, about their learning needs and preferences. In this case, the cancellation of a widely used educational resource due to budget constraints highlighted these communication gaps. Although the library made efforts to gather feedback, only a small portion of students engaged, leaving much of the demand unvoiced. Recognizing the impact on their learning, students took initiative by organizing themselves, collecting evidence, and presenting a cohesive, professional proposal. **Outcomes:** While not always possible, this series of events has created better partnerships with key stakeholders like students. Channels of communication have been established, and students are more willing to communicate with library staff about their educational wants and needs and see the library as a partner in their education. **Discussion:** This project details each step via flowchart over the course of nineteen months, efforts from library staff, students, and others which not only resulted in the reinstatement of the original resource but also led to the addition of a new educational tool to support learning and exam preparation. It demonstrates the significant influence that self-organization, collaboration, and a unified student voice can have in shaping academic resources, bridging communication gaps, and creating positive, lasting change in a library setting.

# Abstract: PP11. Redefining the role of the information professional: our involvement with a distributed undergraduate medical education program

Jane Jun^1^, Kristy Hancock^2^, Kristina McDavid^3^

^1^University of British Columbia Okanagan, ^2^University of Northern British Columbia, ^3^University of British Columbia Vancouver

**Introduction:** The University of British Columbia’s undergraduate medical program uses a distributed education model. The program encompasses the Southern Medical Program, the Vancouver-Fraser Medical Program, the Island Medical Program, and the Northern Medical Program (NMP), and each distributed site has a liaison medical librarian. In 2011, the NMP librarian and the NMP’s Regional Associate Dean “decided to expand the librarian’s role to include [Problem Based Learning] tutoring” (Fyfe & Payne, p. 163). Together, they published a paper that explores the effectiveness of this decision, as well as the value of having a librarian in this role. **Description:** This poster explores the growth in the role of the medical librarians across the distributed undergraduate medical program since 2011 and the ways that we currently support undergraduate medical students. Examples include teaching in the Case-Based Learning (CBL) and the Foundations of Scholarship courses and providing support and leadership with Flexible Enhanced Learning and knowledge synthesis projects. **Outcomes/Discussion:** We discuss opportunities, challenges, and future directions related to serving as liaison librarians for the undergraduate medical program. As suggested by Fyfe and Payne, “[o]ne future direction would be exploring if having a librarian as a Problem Based Learning [now known as CBL] tutor changes medical students’ future scope of practice and how they interact with their future patients” (Fyfe, p. 166).
